# Technology-based interventions for tobacco and other drug use in university and college students: a systematic review and meta-analysis

**DOI:** 10.1186/s13722-015-0027-4

**Published:** 2015-02-24

**Authors:** Amelia Gulliver, Louise Farrer, Jade KY Chan, Robert J Tait, Kylie Bennett, Alison L Calear, Kathleen M Griffiths

**Affiliations:** National Institute for Mental Health Research, The Australian National University, Canberra, Australia; School of Psychology, University of New South Wales, Sydney, Australia; National Drug Research Institute, Faculty of Health Sciences, Curtin University, Perth, Australia

**Keywords:** Systematic review, Meta-analysis, Technology, Intervention, Universities, Students, Drug use, Tobacco

## Abstract

**Background:**

University students have high levels of tobacco and other drug use, yet they are unlikely to seek traditional care. Technology-based interventions are highly relevant to this population. This paper comprises a systematic review and meta-analysis of published randomized trials of technology-based interventions evaluated in a tertiary (university/college) setting for tobacco and other drug use (excluding alcohol). It extends previous reviews by using a broad definition of technology.

**Methods:**

PubMed, PsycInfo, and the Cochrane databases were searched using keywords, phrases, and MeSH terms. Retrieved abstracts (n = 627) were double screened and coded. Included studies met the following criteria: (1) the study was a randomized trial or a randomized controlled trial (RCT); (2) the sample was composed of students attending a tertiary (e.g., university, college) institution; (3) the intervention was either delivered by or accessed using a technological device or process (e.g., computer/internet, telephone, mobile short message services [SMS]); (4) the age range or mean of the sample was between 18 and 25 years; and (5) the intervention was designed to alter a drug use outcome relating to tobacco or other drugs (excluding alcohol).

**Results:**

A total of 12 papers met inclusion criteria for the current review. The majority of included papers examined tobacco use (n = 9; 75%), two studies targeted marijuana use (17%); and one targeted stress, marijuana, alcohol, and tobacco use. A quantitative meta-analysis was conducted on the tobacco use studies using an abstinence outcome measure (n = 6), demonstrating that the interventions increased the rate of abstinence by 1.5 times that of controls (Risk Ratio [RR] = 1.54; 95% Confidence Interval [CI] = 1.20–1.98). Across all 12 studies, a total of 20 technology-based interventions were reviewed. A range of technology was employed in the interventions, including stand-alone computer programs (n = 10), internet (n = 5), telephone (n = 3), and mobile SMS (n = 2).

**Conclusions:**

Although technological interventions have the potential to reduce drug use in tertiary students, very few trials have been conducted, particularly for substances other than tobacco. However, the improvement shown in abstinence from tobacco use has the potential to impact substantially on morbidity and mortality.

**Electronic supplementary material:**

The online version of this article (doi:10.1186/s13722-015-0027-4) contains supplementary material, which is available to authorized users.

## Background

The use of tobacco and other drugs account for almost 5 percent of the global burden of disease in terms of disability-adjusted life years [1], with the prognosis that tobacco use will result in one billion deaths in the 21st century [2]. In addition, substance use disorders are at their peak in young people aged 16 to 25 years [3], at a time when many young people are attending university [4]. A recent study demonstrated that the prevalence of tobacco use in college students was high, with 26.2 percent of students using any tobacco product in the 30 days prior to sampling [5]. In this study [5], smoking cigarettes was the most common method of tobacco use, with 18.6 percent of students smoking cigarettes in the previous 30 days. Other nonalcohol substance use is also prevalent, with a recent study indicating that 9.4 percent of first-year students met criteria for cannabis use disorder [6]. Drug use in young people is typically initiated at the age of, or just prior to, commencement of study at university. For example, in Australia, the mean age of initiation of tobacco use is 16 years and of cannabis is 18.5 years [7]. Early intervention for substance use offers the potential to prevent the development of clinically significant problems. The university setting is therefore an ideal environment in which to provide both broad-scale preventative and treatment approaches [8] for tobacco and other drug use disorders in this group.

The provision of screening and brief intervention has the potential to reduce substance use among university students [9]. However, the high clinical load often experienced at university health clinics may limit the feasibility of face-to-face screening and intervention [10]. Students may also be reluctant to seek help from counseling centers in person [11], with Australian research indicating that young people are particularly unlikely to seek help for drug and alcohol use disorders [12]. A prevalence study conducted in the United States found that rates of substance use were similar between students and nonstudents, but that students who experienced substance use disorders (including alcohol) were less likely to seek help for these problems than young adults in the community (OR = 0.52; 95% CI = 0.30–0.90) [13]. As a result, very few university students will access appropriate care [14] for tobacco and other drug use problems.

Barriers to treatment for substance use problems include cost, difficulty accessing facilities, and stigmatization [15]. The development of online interventions has the potential to circumvent these barriers and to provide a higher level of scalability at minimal marginal cost per user [16]. This is important in the university context, where traditional campus mental health services [17] can be more time consuming for the therapist and less cost-effective than distal interventions [18]. An important benefit of online interventions is “24/7” availability, allowing access either at times of high motivation to change behavior or during periods of increased risk of relapse [19,20]. Publically delivered telephone interventions also utilizing additional methods such as coaching and quit packs, are also often accessible outside regular work hours for this purpose [e.g., Quit for Life, USA (24 hours), Quitline, Australia (8 am–8 pm, Monday–Friday). Additionally, technology-based interventions are easily disseminated, relatively inexpensive, and highly relevant to university populations who are familiar with the use of the internet for health-related problems, particularly information-seeking [21-23].

Computer-delivered interventions appear promising in reducing symptoms of other types of mental health problems [24] as well as for alcohol use [25] in university populations. Previous reviews have examined internet/computer-based tobacco interventions [26-29] or other drug use interventions with college students [30] and in schools [31]. In addition, previous studies have employed a narrow definition of technology confined to the internet or computers and excluded other types of technology (e.g., telephone). Therefore, no reviews have examined both tobacco and other drugs and used a broad definition of technology, including both the internet and other types of technology (e.g., SMS, telephone). The current study systematically reviewed published randomized trials of technology-based interventions evaluated in a tertiary setting for tobacco and other drug use (excluding alcohol).

## Methods

### Search methodology

The PubMed, PsycInfo, and Cochrane Library databases were searched in September 2013 using the keywords, phrases, and MeSH terms presented in Additional file 1. These terms represented three broad concepts: the intervention aim (tobacco/other drug use); population (students enrolled in a tertiary institution – “university” or “college”); and modality (technology such as the internet, telephone, etc.). The keywords, MeSH terms, and phrases pertaining to the first concept (tobacco/other drug use) were derived from the International Classification of Diseases (ICD-10) list of mental disorders, from the National Health and Medical Research Council (Australia, NHMRC) key words for mental health research, as well as additional terms identified by the researchers. The terms relating to the concepts of tertiary students and technology were used in a previous review undertaken by the present authors [24].

### Study selection

**Figure 1:**
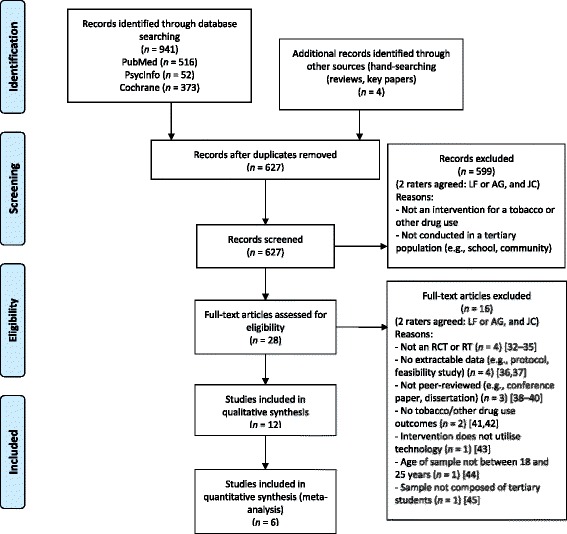
**Study identification flow diagram.**

Figure 1 presents a flow chart describing the study identification process. After the removal of duplicates, the database search yielded 627 English-language abstracts. Table 1 presents the inclusion criteria for each stage of screening. At Stage 1, 627 abstracts were screened by two raters (LF or AG, and JC). Study abstracts that were considered potentially relevant by both raters were retained. Those that were identified as relevant by only one rater were rescreened by both raters according to the inclusion criteria. Following this process, abstracts that both raters considered relevant were retained. The remaining abstracts were discussed by the two raters, and relevant abstracts were mutually agreed upon following this discussion. Through this process, a total of 24 papers were retrieved for a more detailed evaluation (Stage 2). An additional four papers were located by hand-searching the reference lists of papers from the initial 24 identified papers as well as reviews located through the original 941 abstracts. A total of 28 papers were retained for Stage 2 coding and were subject to a stricter set of inclusion criteria (see Table 1). At Stage 2, studies that were considered relevant by both raters were retained, and those that were identified as relevant by only one rater were rescreened by a third rater (LF or AG). A total of 16 studies were excluded at this stage. The reasons for exclusion at this stage were that the study: (1) was not an RCT or randomized trial (n = 4) [32-35]; (2) contained no extractable data (e.g., protocol, feasibility studies) (n = 4) [36,37]; (3) was not peer-reviewed (e.g., conference paper, dissertation) (n = 3) [38-40]; (4) did not contain tobacco/other drug use outcomes (n = 2) [41,42]; (5) intervention did not utilize technology (n = 1) [43]; (6) sample age was not between 18 and 25 years (n = 1) [44]; and (7) sample was not composed of tertiary students (n = 1) [45]. Following this process, 12 papers were judged relevant and included in the current study.

**Table 1:** **Inclusion criteria**

***Stage 1 inclusion criteria***	
1	The study investigated an intervention for tobacco or other drug use.
2	The intervention was either disseminated via or accessed using a technological device (e.g., computer, smart phone, telephone) or process (e.g., e-mail, internet, SMS, video).
3	The study was conducted in a tertiary (university/college) setting with students or young people.
4	The article was in English.
***Stage 2 Inclusion criteria***	
1	Study design – the study was a randomized controlled trial (RCT) or a randomized trial (RT, i.e. an equivalence trial).
2	Recruitment population – the sample was composed of students attending a tertiary institution (e.g., university, college).
3	Intervention type – the intervention (or some portion of the intervention; e.g., reminder or follow-up contact) was either delivered by or accessed using a technological device or process (internet, telephone, video). Studies that used technology to conduct screening or outcome measurement only (that is not considered part of the intervention) did not satisfy this criterion.
4	Age – the age range of the sample was between 18 and 25 years, OR the mean age of the sample was between 18 and 25 years (including up to 25.9 years).
5	Intervention focus – the intervention was designed to alter a drug use outcome relating to tobacco or other drug use and excluding alcohol (e.g., abstinence, intentions).

#### Data extraction

Included studies were each coded by two raters (LF or AG, and JC) using a preformulated rating sheet. Detailed information on the data coded as well as coding categories and study quality is presented in Additional file 2. Study quality was assessed using the risk-of-bias criteria proposed by the Cochrane Effective Practice and Organisation of Care Group (EPOC [46]). Mrazek & Haggerty’s [47] framework was used to code intervention type as determined by the target population, and amount of human contact was coded based on categories identified by Newman and colleagues [48].

#### Data analysis

Descriptive statistics are provided for the 12 included studies. In addition, a meta-analysis of a subset of studies targeting tobacco use was conducted with RevMan [49]. Given that abstinence is regarded as the benchmark for smoking cessation interventions [50], the meta-analysis examined tobacco interventions that used abstinence as an outcome measure and included a control group (n = 6). As per Cochrane guidelines [51], data from a comparison intervention (acting as a second control) in a multi-armed trial (“One Step at a Time”) [52] was combined with the control group data. There were insufficient studies with comparable outcome variables to conduct a meta-analysis for other substances (marijuana). Given the heterogeneity previously reported [53], a random effect model was chosen for the analysis. This assumes the true effect varies from study to study and estimates the mean effect on the random distribution of effects [54]. Statistical heterogeneity was assessed with *I*^*2*^, which is the percentage of variation across studies that is due to heterogeneity rather than to chance. Values of *I*^*2*^ of less than 25 percent are considered low [55], and between 30 and 60 percent may represent moderate heterogeneity [51]. In the present study, *I*^*2*^ was considered low to moderate (32%), although the limited number of studies identified (less than 10) precluded subsample analyses to search for covariates [56]. In addition, to facilitate interpretation of studies that were not included in the meta-analysis, Cohen’s *d* effect sizes were calculated where it was not provided by the study authors, using mean post-intervention and follow-up scores and standard deviations for each intervention group and the control group. Effect sizes for binary outcomes were also expressed as Cohen’s *d* using the formula proposed by Lipsey and Wilson [57]. Negative effect sizes indicate that the control group outperformed the intervention group. Where studies conducted both intention-to-treat (ITT) and completer analyses, results pertaining to the ITT analyses were reported.

The present review conforms to the PRISMA statement [58]. A PRISMA checklist is provided in Additional file 3.

## Results

### Study characteristics

The characteristics of the included studies (n = 12) are presented in Additional file 4. Studies targeted tobacco (n = 9; smoking; n = 8 [52,59-65]; spit tobacco, n = 1) [66]; marijuana, (n = 2) [67,68]; and a multi-targeted study examining stress, marijuana, alcohol, and tobacco use (n = 1) [69]. Since several studies had multiple intervention arms, a total of 20 technology-based interventions targeting tobacco and other drug use were examined from the 12 studies.

### Origin

Most studies targeting tobacco (n = 9) were conducted in the United States [59,60,64-66]; two were conducted in The Netherlands [61,62], and one each in Canada [52] and Germany [63]. The two studies targeting marijuana and the multi-targeted study [67-69] were all conducted in the United States.

### Participants and target group

Sample sizes overall ranged from 65 to 517 (median = 223.5). The majority of studies were randomized at the participant level, with two [65,66] randomized at the institution level. The included studies almost exclusively targeted participants using the substance (treatment interventions; 10 of 12 studies; [52,59-66,68]). All tobacco interventions included in the meta-analysis (n = 6) were treatment interventions. There was one universal trial where students were not selected on the basis of substance use [69], and one of the two studies on marijuana specifically targeted students who were *not* using the substance [67].

### Technology employed

The 20 interventions from the 12 included studies utilized a range of technology types, including stand-alone computer programs (n = 10; five studies [61,62,64,65,69]); the internet (n = 5; four studies [59,60,67,68]); telephone (n = 3; two studies [52,66]); and mobile SMS (n = 2 [63]). Tobacco interventions included in the meta-analysis (n = 6) were delivered by computer [64,65], the internet/email [59,60], or telephone [52,66]. The marijuana interventions were both delivered via the internet [67,68], and the multi-targeted study used computers [69].

### Intervention length and delivery

The intervention duration was reported for less than half of the 20 interventions (n = 8; six studies [59,60,63,64,66,67]) and ranged from 15 minutes [59] to 30 weeks [60]. Most (n = 4) of the tobacco interventions included in the meta-analysis reported intervention duration (range as above).

Overall, 10 interventions were delivered on site (i.e., “nondistal,” six studies; [59,61,62,64,65,69]), seven were distal (five studies; [52,60,63,67,68], and three combined distal and nondistal components (two studies; [59,66]).

### Level of human contact

The majority of interventions overall (n = 13; 65.0%; seven studies [61-64,67-69]) were *self-administered,* including all three of the multi-targeted/marijuana interventions [67-69]. However, of the studies included in the meta-analysis, only one was self-administered [64]. The remainder of the interventions in the meta-analysis comprised *predominantly self-help* interventions [52,59,60], *minimal contact* interventions (active involvement of a therapist to a lesser degree than traditional therapy) [59,66], and a *therapist-administered* intervention [65].

### Outcome measures

The majority of the nine tobacco studies used abstinence as their primary outcome measure (n = 6; 66.7%). These were the studies examined in the meta-analysis. Four studies measured 7-day abstinence [52,59,65,66], and one study measured 30-day abstinence [60]. The study targeting spit tobacco [66] used 7-day point-prevalence abstinence with cotinine verification, and one tobacco study did not provide a required duration of abstinence [64]. All studies included in the meta-analysis used self-reported abstinence; two also used additional cotinine verification [59,65], and one used cotinine verification alone [66].

The other studies targeting smoking used number of cigarettes smoked per day [63], intention to quit [62], and “quitting activity” [61] as outcome measures. Finally, the two marijuana studies used a measure of any use of marijuana in the past month [67] or the number of days marijuana was used during the previous 90 days [68], and the multi-targeted study used the participant’s intentions to use marijuana in the next 6 months [69]. The latter study also measured intentions to use cigarettes and alcohol during this period.

### Study quality

Overall, almost all studies employed control conditions, except for one study that compared four interventions [62]. Of the six tobacco RCTs included in the meta-analysis, most used usual care controls [52,59,60,65], one used attention placebo [64], and one (spit tobacco) [66] used a no-intervention control. The remaining RCTs (n = 5) used usual care control conditions [61,69] or a no-intervention control [63,67,68].

Table 2 displays assessment details of each study using EPOC quality rating criteria. The majority (n = 10) of the 12 studies overall employed ITT analyses, with five of these using the baseline-observation-carried-forward-approach [52,59,60,63,66]. Only one study included in the meta-analysis (n = 6) did not employ an ITT analysis [64].

**Table 2:** **Quality rating criteria met by each study using the Cochrane Effective Practice and Organisation of Care (EPOC) guidelines**

**Study author and year**	**Allocation sequence**	**Allocation concealment**	**Baseline measurements - participants**	**Baseline characteristics - providers**	**Incomplete data addressed**	**Knowledge of allocation**	**Contamination protected**	**Selective outcome reporting**	**Free of other bias**
*Smoking tobacco* (n *=* 8)									
Haug et al. (2009) [63]	✓	▪	✓	✓	✓	▪	✓	✓	✓
Travis & Lawrance (2009) [52]*	▪	▪	✓	▪	✓	▪	✓	✓	✓
O’Neill et al. (2000) [64]*	▪	▪	▪	▪	▪	▪	✓	✓	✓
An et al. (2008) [60]*	✓	▪	✓	▪	✓	▪	✓	✓	✓
Dijkstra (2005) [61]	▪	▪	▪	▪	▪	▪	✓	✓	✓
Abroms et al. (2008) [59]*	▪	▪	✓	✓	✓	▪	✓	✓	✓
Dijkstra & Ballast (2012) [62]^†^	▪	▪	▪	✓	✓	▪	✓	✓	✓
Prokhorov et al. (2008) [65]*	▪	▪	✓	✓	✓	▪	✓	✓	✓
*Spit tobacco* (n *=* 1)									
Masouredis (1997) [66]*	▪	▪	✓	▪	✓	▪	✓	✓	✓
*Marijuana* (n *=* 2)									
Lee (2010) [68]	✓	▪	✓	✓	✓	▪	✓	✓	✓
Elliott (2012) [67]	▪	▪	✓	✓	✓	▪	✓	✓	✓
*Stress, marijuana, alcohol, and tobacco* (n *=* 1)									
Moore et al. (2012) [69]	✓	▪	▪	▪	✓	▪	✓	✓	✓

Note: *Studies included in the meta-analysis; ^†^= No control group (randomized trial).

### Intervention efficacy

#### Tobacco

**Figure 2:**
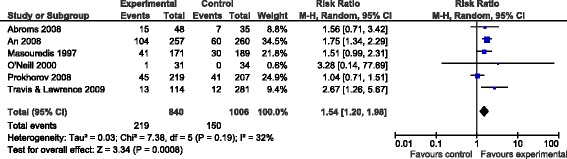
**Data and weights for studies included in the meta-analysis for technology-based tobacco interventions.**

Interventions that included a control condition as well as abstinence measure were included in the meta-analysis (n = 6) [52,59,60,64-66]. Figure 2 displays the data and weights included in the meta-analysis for the technology-based tobacco interventions on abstinence. Overall, the interventions increased the rate of abstinence by 1.5 times that of controls (RR = 1.54; 95% CI = 1.20, 1.98). Heterogeneity was low to moderate, with *I*^*2*^ = 32 percent. Results were similar when the spit tobacco intervention was excluded, and only interventions examining smoking were examined [66]) (RR = 1.57; 95% CI = 1.11, 2.22) with *I*^*2*^ at 46 percent.

For the two tobacco RCTs not included in the meta-analysis, the first found a significant positive effect of two interventions for quitting activity, which included those participants who had made any attempt to quit. The effective interventions were brief education consisting of 800 words of either “personalized” (using the person’s name, years smoked) or “tailored” (providing feedback using scores from pre-test) information [61] (*d* = unable to calculate). In this study, the intervention that was not effective contained adapted information (tailored only for certain characteristics; e.g., gender, age). The second study did not find a significant positive effect (*d* = −0.12, −0.03) for two 3-month, individualized, SMS feedback programs (one including two extra feedback assessments) compared with a no-intervention control [63].

#### Other drugs

The effect sizes at post-intervention for interventions targeting marijuana were 0.38 [68] and −0.01 [67], and 0.28 for the multi-targeted intervention (intentions, cigarettes = 0.29; intentions, marijuana = 0.27; n = 1) [69]. Only one intervention out of the three other drug use studies found a positive effect. The multi-targeted intervention [69] comprising a brief computer program plus feedback (which did not measure abstinence) found a positive outcome for intention to smoke cigarettes, but not for intention to use marijuana for the intervention group compared with a usual care control condition. Neither of the marijuana interventions [67,68] consisting of brief web- or computer-based personalized feedback programs, was effective at reducing [68] or preventing (in abstainers) [67] marijuana use for participants in the intervention condition compared with no-intervention control conditions.

## Discussion

The current systematic review identified 12 randomized trials detailing 20 technology-based interventions targeting tobacco or other drug use in tertiary students. The majority of papers (n = 9) targeted tobacco use, with eight of these targeting smoking. The meta-analysis conducted on the subset of tobacco studies reporting abstinence demonstrated that the interventions increased the rate of abstinence by 1.5 times that of controls. The duration of this abstinence varied between 7 and 30 days (one study did not report required duration). Only two studies targeted marijuana use, and one study used a multifaceted approach targeting stress, marijuana, alcohol, and tobacco use. Outcomes for the marijuana and multi-targeted study were mixed. Overall, three-quarters of the interventions were delivered using computers or the internet, with a minority using telephone or SMS technology.

The current review and meta-analysis indicates that technology-based interventions are promising for reducing tobacco use in tertiary students. Our findings are similar to those by Myung et al. [26] reporting on 22 internet- or computer-based smoking cessation programs that yielded an abstinence rate 1.5 times higher than the control group (RR, 1.44; 95% CI, 1.27–1.64). Considering internet-based interventions alone, Civljak and colleagues [53] reported benefits, especially where the information is appropriately tailored and employs frequent automated contacts. However, the internet-based programs did not show consistent effects. Overall, this suggests the use of either proximal technology-based interventions or more intensive/tailored internet programs.

The majority of interventions for tobacco use in the meta-analysis were compared with usual care control conditions, indicating that additional, tailored content may increase abstinence in this group as suggested above. Indeed, the addition of tailoring to several usual care (standard) interventions [52,59], specifically to the young adult age group, was associated with moderate effects. The intervention with the strongest effect size was the most intensive, including a 30-week program with access to a website plus personalized follow-up emails from peer coaches [60]. This intervention also included a chance to win a $3000 prize for all participants. This raises the possibility that more intensive interventions may result in higher abstinence rates. Nevertheless, even brief written information was found to be effective in some studies [52,61], albeit not others [64]. While age-tailored content appears useful, further research is needed to determine which other information is most effective in increasing abstinence in this population.

The two marijuana interventions were not effective at reducing or preventing marijuana use [67,68]. The results of a recent review were inconclusive regarding the effectiveness of prevention programs for marijuana in young people [70]. The authors attributed this in part to the poor methodology of the included papers, where approximately half of the included 25 studies failed to deal appropriately with attrition or provide sufficient data to calculate effect sizes. In contrast, the quality of the two marijuana studies in the current review was relatively high. Nevertheless, neither reported a positive outcome. The authors of these papers suggest that future studies should target certain groups of students (i.e., those with family history of the substance, and those with higher contemplation of changing their marijuana use) [68], as well as nonusers, and examine longer-term follow-up data (>1 month), where preventative effects may more likely be detected [67]. Additionally, a previous paper suggested it was important to take into account participant preference for technology type in marijuana interventions (e.g., telephone vs. web-based [71]).

Less than half of the papers included in the present review examined interventions that were delivered distally. Given that distally-delivered internet interventions may be perceived as less stigmatizing than traditional approaches to care [18,72] and that smoking [73] and drug use [74] are stigmatized among young people, internet interventions may hold greater appeal for students who may be concerned with stigma associated with seeking services on campus [75]. Therefore, it is important that further studies investigate distal methods, such as the internet, to deliver interventions targeting tobacco and other drug use. In addition, only one study using mobile phones was identified in the current study. Although it was not found to be effective, more research on this device is needed. Young people aged 16–34 years are highly familiar with mobile technology and are the most likely age group to own a smart phone [76]. Therefore, further investigation into the delivery of interventions for this group using mobile phone applications is particularly important, as a systematic review examining the use of mobile phones for smoking cessation in the general population reported sustained benefits for this approach [77].

The majority of included studies targeted smoking, which is unsurprising given the high prevalence of smoking [5] compared to other types of drug use [6]. Tobacco use is the leading cause of preventable illness and premature death across the world [78]. Given the high prevalence of smoking and its associated morbidity/mortality, even small improvements in cessation could have a major impact on public health. Despite this and the relevance and applicability of technology-based approaches to this population [21-23], very few technology-based programs for tobacco use, especially prevention programs, were identified in the current review. Further technology-based interventions on tobacco use are needed in this at-risk group.

Abstinence remains the gold standard of smoking cessation interventions, as exemplified by the “Russell Standard,” which recommends continuous abstinence for 6 months as the key outcome for cessation trials [50]. None of the studies reported against such a stringent criterion. Four [59,60,64,65] reported an abstinence measure at 6 months, but all used short-term abstinence (e.g., 7 days) at this time point. Some studies in the present review chose to measure other outcomes such as intentions to quit, particularly for nontreatment interventions. Interventions for other substances may take a “harm reduction” approach [19]. Under this approach, reducing the frequency of cannabis use, as sought by Lee and colleagues [68] would be regarded as a credible outcome.

The majority of studies utilized self-report methods, although some combined these measures with chemical tests using cotinine [59,65] or carbon monoxide testing [60]. However, many studies still rely on self-report. For example, An et al. [60] used carbon monoxide testing, yet used self-report 30-day abstinence as the primary outcome variable, with the authors suggesting that their testing equipment was not sensitive enough to detect occasional smoking [60]. Chemical detection methods can be problematic. Previous research [79,80] has documented high levels of inconsistency between self-reported behavior and biological verification. More sophisticated methods of detecting residual chemicals from tobacco smoking should be investigated [60].

The studies displayed some methodological problems. All RCTs failed to report sufficient information about randomization concealment, and almost two-thirds failed to report adequate randomization methods. Encouragingly, the majority of studies employed ITT analyses, including almost all of the studies included in the meta-analysis. However, no studies met the criterion of blinding the “outcome assessors” to the allocated interventions. This is because in psychological interventions using self-report, the participant is the outcome “assessor” and is not able to be blinded to their condition [81]. Additionally, as pointed out by Farrer et al. [24], study quality criteria could be refined to more accurately assess the quality of internet-based research.

### Limitations

There are limitations to the present review that require consideration. First, given the present review sought to evaluate the evidence relevant to interventions in tertiary student populations, the results may not be applicable more broadly to the general population. A second limitation is that the sample size of studies included in the meta-analysis and the detected effect size were relatively small. Additionally, the current review searched three databases, and it is possible that relevant journals may not be indexed by these databases. However, hand-searching previous reviews and key papers was utilized to address this issue [82]. Finally, it is possible that the incorporation of published papers only may have biased the review, given that publication may be biased towards papers with positive outcomes [83]. Likewise, this also applies to the inclusion of English-language studies only.

## Conclusions

Technology-based interventions for tobacco cessation show promise in the tertiary student population. Despite the serious negative consequences associated with tobacco and other drug use, there is a paucity of research targeting these conditions using technology-based interventions in university students.

## Additional files

Additional file 1:
**Search terms.**
Additional file 2:
**Data extraction coding notes.**
Additional file 3:
**PRISMA checklist.**
Additional file 4:
**Table of included studies.**
