# Supplementary material for: Technology-based interventions for tobacco and other drug use in university and college students: a systematic review and meta-analysis
Source: Addict Sci Clin Pract. 2015 Feb 24;10(1):5. doi: 10.1186/s13722-015-0027-4 (PMC4422468; doi:10.1186/s13722-015-0027-4)
Supplement: Additional file 1: — Search terms. [file 13722_2015_27_MOESM1_ESM.docx]

**Additional file 1: Search strategy**

**Pubmed 02/09/13**

| #4 | Search (#1 AND #2 AND #3) Filters: Clinical Trial; Clinical Trial, Phase III; Controlled Clinical Trial; Randomized Controlled Trial; Clinical Trial, Phase IV; Clinical Trial, Phase I; Clinical Trial, Phase II | 516 | |
| --- | --- | --- | --- |
| #3 | Search (computer OR computer-based OR cyber OR cyberspace OR electronic OR “electronic mail” OR email OR e-mail OR internet OR internet-based OR net OR online OR virtual OR web OR web-based OR webbased OR “world wide web” OR www OR phone OR telephone OR “smart phone” OR “cell phone” OR iphone OR sms or “short message service” OR texting OR “text message” OR “text messaging” OR mobile OR “mobile phone” OR ipad OR tablet OR “smart device” OR digital OR “personal digital assistant” OR pda OR CD-ROM OR "computers"[MeSH Terms] OR "electronics"[MeSH Terms] OR "electronic mail"[MeSH Terms] OR "internet"[MeSH Terms] OR "telephone"[MeSH Terms] OR "text messaging"[MeSH Terms] OR "cd-rom"[MeSH Terms]) | 1229457 | |
| #2 | Search (“addiction” OR “alcohol dependence” OR “substance use” OR “substance abuse” OR intoxication OR harmful use OR withdrawal OR alcohol OR opioid* OR cannabinoids OR cannabis OR sedatives OR hypnotics OR cocaine OR stimulants OR caffeine OR hallucinogens OR tobacco OR “volatile solvents” OR “drug use” OR “drug abuse” OR “drug dependen*” OR “drug relapse” OR “drugs of dependence” OR “opioid dependen*” OR “smoking cessation” OR “smoking intervention” OR “tobacco control” OR “tobacco dependen*” OR “tobacco use” OR amphetamine OR “crystal meth*” OR GHB OR heroin OR ice OR marijuana OR MDMA OR methamphetamine* OR polysubstance OR phencyclidine[Title/Abstract]) | 1460855 | |
| #1 | Search (university[Title/Abstract] OR universities[Title/Abstract] OR university-based[Title/Abstract] OR tertiary[Title/Abstract] OR college[Title/Abstract] OR colleges[Title/Abstract] OR student[Title/Abstract] OR students[Title/Abstract] OR undergraduate[Title/Abstract] OR undergraduates[Title/Abstract] OR dorm[Title/Abstract] OR dorms[Title/Abstract] OR fraternity[Title/Abstract] OR fraternities[Title/Abstract] OR sorority[Title/Abstract] OR sororities[Title/Abstract] OR academic[Title/Abstract] OR academics[Title/Abstract] OR academia[Title/Abstract] OR scholar[Title/Abstract] OR scholars[Title/Abstract] OR "students"[MeSH Terms] OR "universities"[MeSH Terms]) | 546939 |  |

**PsycINFO (02/09/13)**

| 1 | exp Community Colleges/ or exp Colleges/ | 11265 |
| --- | --- | --- |
| 2 | exp Students/ | 175477 |
| 3 | (university or universities or university-based or tertiary or college or colleges or student or students or undergraduate or undergraduates or dorm or dorms or fraternity or fraternities or sorority or sororities or academic or academics or academia or scholar or scholars).ab,ti. | 540445 |
| 4 | 1 or 2 or 3 | 585431 |
| 5 | (addiction or "alcohol dependence" or "substance use" or "substance abuse" or intoxication or "harmful use" or withdrawal or alcohol or opioid$ or cannabinoids or cannabis or sedatives or hypnotics or cocaine or stimulants or caffeine or hallucinogens or tobacco or "volatile solvents" or "drug use" or "drug abuse" or "drug dependen$" or "drug relapse" or "drugs of dependence" or "opioid dependen$" or "smoking cessation" or "smoking intervention" or "tobacco control" or "tobacco dependen$" or "tobacco use" or amphetamine or "crystal meth$" or GHB or heroin or ice or marijuana or MDMA or methamphetamine$ or polysubstance or phencyclidine).ab,ti. | 204561 |
| 6 | (computer or computer-based or cyber or cyberspace or electronic or "electronic mail" or email or e-mail or internet or internet-based or net or online or virtual or web or web-based or webbased or "world wide web" or www or phone or telephone or "smart phone" or "cell phone" or "cellular phone" or iphone or sms or "short message service" or texting or "text message" or "text messaging" or mobile or "mobile phone" or ipad or tablet or "smart device" or digital or "personal digital assistant" or pda or CD-ROM or technology or technologies or technological).ab,ti. | 190868 |
| 7 | 4 and 5 and 6 | 1848 |
| 8 | limit 7 to ("2000 treatment outcome/clinical trial" and ("0100 journal" or "0110 peer-reviewed journal")) | 52 |

**Cochrane Library (02/09/13)**

1. ("addiction" or "alcohol dependence" or "substance use" or "substance abuse" or intoxication or "harmful use" or withdrawal or alcohol or opioid* or cannabinoids or cannabis or sedatives or hypnotics or cocaine or stimulants or caffeine or hallucinogens or tobacco or "volatile solvents" or "drug use" or "drug abuse" or "drug dependen*" or "drug relapse" or "drugs of dependence" or "opioid dependen*" or "smoking cessation" or "smoking intervention" or "tobacco control" or "tobacco dependen*" or "tobacco use" or amphetamine or "crystal meth*" or GHB or heroin or ice or marijuana or MDMA or methamphetamine* or polysubstance or phencyclidine):ti,ab,kw in Trials (45975)
2. (computer or computer-based or cyber or cyberspace or electronic or "electronic mail" or email or e-mail or internet or internet-based or net or online or virtual or web or web-based or webbased or "world wide web" or www or phone or telephone or "smart phone" or "cell phone" or "cellular phone" or iphone or sms or "short message service" or texting or mobile or "mobile phone" or ipad or tablet or "smart device" or digital or "personal digital assistant" or pda or CD-ROM or technology or technologies or technological):ti,ab,kw in Trials (42658)
3. (university or universities or university-based or tertiary or college or colleges or student or students or undergraduate or undergraduates or dorm or dorms or fraternity or fraternities or sorority or sororities or academic or academics or academia or scholar or scholars):ti,ab,kw in Trials (30924)
4. #1 AND #2 AND #3 (373)
