# Supplementary material for: Technology-based interventions for tobacco and other drug use in university and college students: a systematic review and meta-analysis
Source: Addict Sci Clin Pract. 2015 Feb 24;10(1):5. doi: 10.1186/s13722-015-0027-4 (PMC4422468; doi:10.1186/s13722-015-0027-4)
Supplement: Additional file 2: — Data extraction coding notes. [file 13722_2015_27_MOESM2_ESM.docx]

## Additional file 2: Data extraction coding notes

Data coded: The included studies were each coded by two raters (LF or AG, and JC). Data coded comprised the following: author name, country where the study was conducted, participant characteristics and recruitment method, criteria to be involved in the study, description of the intervention, intervention type (target group) and the theoretical underpinnings, the technology used, whether or not the intervention was distal, amount of human contact involved in the intervention, whether or not intention-to-treat (ITT) analysis was employed, an overall quality rating for the study, the primary outcome measure for the study, measurement occasions, whether or not the intervention was significantly superior to the control at each measurement occasion, and finally, Cohen’s *d* effect sizes for the difference between each intervention and the control group at each measurement occasion.

Coding of study outcomes: Descriptive information regarding whether the study reported a significant difference between the intervention and control group at post-intervention and follow-up time points was reported. This information was reported for the primary outcome measure(s) as specified by the authors. In the event that a primary outcome was not specified, only the first outcome that was described in the measures section of the paper was reported. In addition, if the primary outcome was not an abstinence or drug use outcome (i.e., intention to quit), then this was noted, and only the abstinence or drug use data were recorded. If no abstinence data were reported, the primary outcome was recorded instead.

Intervention type (target group): Mrazek & Haggerty’s [47] framework was used to code intervention type as determined by the target population. *Universal* interventions were those without a target group (screening for drug use not necessary), *Selective* interventions were those that targeted individuals at risk of a drug use problem (involved screening); *Indicated* interventions were those that targeted individuals displaying symptoms of a drug use problem (involved screening), and *Treatment* programs were those that targeted individuals currently using the drug. An additional category (“nonusers”) that included the specific targeting of those not using the substance (as opposed to Universal, which included all participants, or Selective/Indicated, which involved a level of risk for drug use) was added to the coding.

Amount of human contact was coded based on categories identified by Newman and colleagues [48]: (a) *SA: self-administered* therapy (therapist/human contact for assessment, at most); (b) *PSH*: *predominantly self-help* (therapist/human contact beyond assessment for periodic check-ins, teaching clients how to use the self-help tool, and/or for providing the initial therapeutic rationale. If any assistance in the use of therapeutic tools was provided, it did not involve more than 1.5 hours of the therapist’s/human’s time); (c) *MC: minimal-contact therapy* (active involvement of a therapist/human, though to a lesser degree than traditional therapy for this disorder; included any treatment in which the therapist/human assisted the client in the application of specific therapy techniques and that involved more than 1.5 hours of the therapist's/human’s time); and (d) *TA: predominantly therapist-administered treatments* (clients had regular contact with a therapist/human for a typical number of sessions, but the study attempted to determine whether the use of a self-help tool augmented the impact of the standard therapy). In studies where reminders were provided and no human involvement or tailoring was reported, the reminders were considered to be automated, and the study was categorized as self-administered. Interventions were considered to be distal if they “travelled” to the recipient, rather than the recipient being required to come to the site to participate in the intervention.

Study quality was assessed using the risk of bias criteria proposed by the Cochrane Effective Practice and Organisation of Care Group (EPOC, [46]), a measure designed to assess potential sources of bias for studies involving a control group. Items were designed to measure bias relating to inadequate random allocation sequence and allocation concealment, differences in baseline outcome measurements and characteristics, inadequate treatment of missing outcome data, researcher knowledge of allocated interventions, contamination between the conditions, and selective outcome reporting as well as any other risk of bias. A score of 1 was awarded for each criterion adequately addressed within the paper, with potential scores ranging from 0 to 9 (for quality rating criteria, see Table 1).
