# Supplementary material for: Technology-based interventions for tobacco and other drug use in university and college students: a systematic review and meta-analysis
Source: Addict Sci Clin Pract. 2015 Feb 24;10(1):5. doi: 10.1186/s13722-015-0027-4 (PMC4422468; doi:10.1186/s13722-015-0027-4)
Supplement: Additional file 4: — Table of included studies. [file 13722_2015_27_MOESM4_ESM.docx]

## Additional file 4: Table of included studies

| **Study and**  **country** | **Participants**  **(# randomized)**  **(# analyzed, if not ITT) and recruitment** | **Age**  **(M, SD)**  **sex (%F)** | **Intervention description** | **Criteria and theoretical framework** | **Technology used** | **Distal?** | **Human contact?** | **ITT?** | **Quality rating** | **Primary outcome and**  **measurement points** | **Significant difference reported between intervention groups at time point?** | **Effect size between intervention and the control (Cohen’s *d*)** |
| --- | --- | --- | --- | --- | --- | --- | --- | --- | --- | --- | --- | --- |
| Smoking tobacco (*n* = 8) | | | | | | | | | | | | |
| Haug et al. (2009)  [63]  Germany | *N* = 174, I^1^ = 50, I^2^ = 60, C = 64  Visitors to university cafeteria who were daily smokers and used SMS at least weekly.  *Recruitment:* Participants recruited from university cafeteria | M = NR, C = 25.4, I^1^ = 25.2, I^2^ = 24.3.  SD = NR, C = 4.9, I^1^ = 4.8, I^2^ = 3.8.  F = 57.9% | I^1^ = SMS Coach: 3-month program of weekly SMS-based assessment of smoking status and intention to quit + 1 individualized SMS feedback assessment + access to automated SMS craving support.  I^2^ = I^1^ + 2 extra individualized SMS feedback assessments (3 total).  C = No intervention | Daily smokers  Treatment  Stages of Change | I^1^ = SMS  I^2^ = SMS  C = N/A | I^1^ = Y  I^2^ = Y  C = N/A | I^1^ = SA  I^2^ = SA  C = N/A | Y = BOCF | 7/9 | Number of cigarettes smoked per day (self-report)  Baseline  3 months  3 months – cigarettes smoked per day, M (SD):  I^1^ = 10.2 (6.5)  I^2^ = 9.7 (6.4)  C = 9.5 (5.5) | 3 months:  I^1^ : N  I^2^ : N  Comparison between groups at post-intervention only. | 3 months :  I^1^ : -0.12  I^2^ : -0.03 |
| Travis & Lawrance (2009)  [52]*  Canada | *N* = 395, I^1^ = 114, I^2^ = 137, C = 144.  University students who voluntarily visited a ”Leave the Pack Behind” display or presentation.  *Recruitment:* Visitors to display or present who requested smoking-cessation resources. | M = 21.37  SD = 3.35  F = 50.5% | I^1^ = Smoke\|Quit 2-booklet, self-help program: 1) Smoke precontemplation information booklet, 2) Quit booklet with quit information and research e.g., staying smoke-free, quizzes, CBT exercises, countdown quit plan and relapse prevention strategies.  I^2^ = One Step at a Time: 2 booklet stage-based resource containing similar information to I^1^ + broader range of adult experiences e.g., working, parenting.  C = Usual care quit kit with age-appropriate smoking cessation information including quit leaflet, smoking diary, pharmacological intervention pamphlet, “how to quit smoking” handout, coping with stress booklet, and novelty items (e.g., chewing gum).  All groups received a brief support telephone call from a trained student-peer ~1 month after registration. | Current smokers  Treatment  Stages of Change | I^1^ = Telephone  I^2^ = Telephone  C = Telephone | I^1^ = Y  I^2^ = Y  C = Y | I^1^ = PSH  I^2^ = PSH  C = PSH | Y = BOCF | 5/9 | Smoking cessation (self-report 7-day point-prevalence).  Baseline  3 months  3 months – reported 7-day cessation, N (%):  I^1^ = 13 (11.4)  I^2^ = 4 (2.9)  C = 8 (5.6) | 3 months:  I^1^ : Y  I^2^ : N | 3 months:  I^1^ : 0.15  I^2^ : -0.50  (based on final n reported quitting) |
| O’Neill et al. (2000) [64] *  USA | *N* = 65, I = 31, C = 34 (randomized);  *N* = 65, I = 31, C = 34 (post); *N* = 64, I = 31, C = 34 (1 month); *N* = 61, I = 29, C = 32 (3 months); *N* = 56, I = 27, C = 29 (7 months).  “Lower level” psychology students who were daily smokers.  *Recruitment:* Students in psychology classes completed screening survey and were contacted via telephone if eligible. | M = 19.7  SD = NR  F = 63.1% | I = 3 x sessions of “Smoke Mall” educational computer modules delivered fortnightly over 6 weeks.  C = 3 x sessions of health-related (dietary, hypertension, stress) educational computer modules delivered fortnightly over 6 weeks. | Daily smokers  Treatment  Stages of Change | I = Computer  C = Computer | I = N  C = N | I = SA  C = SA | N | 3/9 | Self-reported abstinence (duration NR)  (primary outcome was motivation to stop smoking)  Baseline  6 weeks (post)  1 month (after post)  3 months (after post)  7 months (after post)  6 weeks (post) – achieved abstinence (duration NR), N (%):  I = 1 (2.9)  C = 0 (0) | 6 weeks: N  1 month: N  3 months: N  7 months: N | 6 weeks:  unable to be estimated*  1 month:  0.16  3 months:  0.26  7 months:  0.09  (based on final % self-reported abstinence)  *(1 person achieved abstinence in intervention group) |
| An et al. (2008) [60] *  USA | *N* = 517, I = 257, C = 260  Undergraduate students who had smoked in the past 30 days and intended to be in school for the following 2 semesters.  *Recruitment:* -internet broad health screening survey sent to 25,000 students. | M = NR, I = 20.1, C = 19.8.  SD = NR, I = 1.6, C = 1.6.  F = 72.9% | I = 20 x weekly visits to RealU intervention website over 30-week period, includes personalized weekly email from peer coach, with correspondence rewarded as an entry for a $50 prize contest. Also includes weekly email invitations to visit website to report on previous week’s habits, quizzes with tailored feedback on smoking and general interest + view a student-authored magazine article (at least 1 from 5 on quitting in college) + $10 gift card incentive for completing the above activities.  C = Email with links to health (e.g., QuitNet.com) and academic resources.  Both interventions received advertisements for a quit and win prize contest with a prize draw of $3000 for remaining abstinent for 1 month. | Smoked cigarettes in the past 30 days  Treatment  No theory identified | I = internet  C = internet | I = Y  C = Y | I = PSH  C = SA | Y = BOCF | 6/9 | Self-reported 30-day abstinence  Baseline  8 weeks  20 weeks  30 weeks  30 weeks – achieved 30-day abstinence, N (%):  I = 104 (40.5)  C = 60 (23.1) | 8 weeks:  N  20 weeks:  N  30 weeks:  Y | 8 weeks:  -0.008  20 weeks:  0.15  30 weeks:  0.45 |
| Dijkstra (2005) [61]  The Netherlands | *N* = 202, I^1^ = 50, I^2^ = 51, I^3^ = 50, C = 51 (Randomized).  *N* = 202 (post), I^1^ = 50, I^2^ = 51, I^3^ = 50, C = 51; *N* = 141 I^1^ = NR, I^2^ = NR, I^3^ = NR, C = NR (4 months).  University students who were daily smokers.  *Recruitment:* Students registered their phone numbers on forms distributed in faculty lounge, and students present in faculty lounge were approached directly. | M = 22.2  SD = 6.5  F = 59.0% | I^1^ = *Personalized* (e.g., referred to participant’s name, years smoked) information about consequences of smoking.  I^2^ = *Adapted* information about consequences of smoking, tailored for certain characteristics only (e.g., gender, sport participation).  I^3^ = *Tailored* *feedback* and information about consequences of smoking using scores from pretest.  C = Standard information about consequences of smoking.  All interventions were 4 x screens of 200 words each. | Daily smokers  Treatment  ELM | I^1^ = Computer  I^2^ = Computer  I^3^ = Computer  C = Computer | I^1^ = N  I^2^ = N  I^3^ = N  C = N | I^1^ = SA  I^2^ = SA  I^3^ = SA  C = SA | N  (No dropout at post only) | 4/9 | Quitting activity – any attempt to quit during the 4-month period (included a yes to any of the following: 7- or 30-day or 4-month point-prevalence abstinence or a yes response to “did you make an attempt to quit since you were in the laboratory?”).  Baseline  4 months  (the primary outcome – “attitudes towards smoking” was measured immediately post-test)  4 months – any attempt to quit, % (N for each group not listed):  I^1^ = 44.7  I^2^ = 28.6  I^3^ = 48.5  C = 22.9 | 4 months:  I^1^ : Y  I^2^ : N  I^3^ : Y | 4 months:  I^1^ : ID  I^2^ : ID  I^3^ : ID |
| Abroms et al. (2008) [59] *  USA | *N* = 83, I = 48, C = 35.  Undergraduate students smoking at least 1 cigarette/day in the past 7 days and were interested in quitting in the next 6 months.  *Recruitment:*  Flyers/advertisements placed in the college newspaper plus a study staff table outside the student center. | M = 19.8  SD = 1.3  F = 54.2% | I = *X-Pack* program: 15-minute in-person counseling session, a self-help kit targeting young adults including guidebook, wallet-sized quit cards, motivating slide rule, cigarette substitutes (gum, putty, toothpicks) + 10-12 x tailored counseling emails, and 4 x emails on and around the participant-selected quit day.  C = *Clearing the Air* program: 15-minute in-person counseling session + a self-help kit targeting a general adult audience. | Smoking at least 1 cigarette per day in each of the past 7 days.  Treatment  No theory identified. | I = Email  C = Email | I = Combined  C = Combined | I^1^ = MC  C = PSH | Y = BOCF | 7/9 | Self-report 7-day abstinence from smoking  Cotinine verification (at 6 months only)  3 months  6 months  3 months – achieved 7-day abstinence, N (%):  I = 15 (31.3)  C = 7 (20.0) | 3 months: N  6 months: N | 3 months: 0.33  6 months: 0.38  (based on final % abstinence). |
| Dijkstra & Ballast (2012) [62]  The Netherlands | *N* = 121, I^1^ = 32, I^2^ = 32, I^3^ = 29, I^4^ = 28.  University students who were smoking on campus.  *Recruitment:* Researchers approached smokers at locations within 2 universities. | M = 22.9  SD = 63.9  F = 75.0% | I^1^ = Standard persuasive messages using ”weak” arguments with poor reasons to encourage intention to quit.  I^2^ = Standard persuasive messages using “strong” arguments with good reasons to encourage intention to quit.  I^3^ = Personalized (using the participant’s name 3 times) persuasive messages using weak arguments with poor reasons to encourage intention to quit.  I^4^ = Personalized (using the participant’s name 3 times) persuasive messages using strong arguments with good reasons to encourage intention to quit.  All interventions were approximately 120 words, presented on a computer screen. | Smokers who were smoking at university locations.  Treatment  Personalization | I^1^ = Computer  I^2^ = Computer  I^3^ = Computer  I^4^ = Computer | I^1^ = N  I^2^ = N  I^3^ = N  I^4^ = N | I^1^ = SA  I^2^ = SA  I^3^ = SA  I^4^ = SA | Y = No dropout | 5/9 | Intention to quit  Baseline  Post (immediate)  (no abstinence measure)  I^1^ = ID  I^2^ = ID  I^3^ = ID  I^4^ = ID | Post (immediate):  I^1^ = I^2^ = I^3^ = I^4^  (main effects of Personalization and argument quality was NS).  Study used pretest scores as covariate (ANCOVA). | Post (immediate):  I^1^ : NC  I^2^ : NC  I^3^ : NC  I^4^ : NC |
| Prokhorov et al. (2008) [65] *  USA | *N* = 426, I = 219, C = 207 (participants)  14 campuses randomized, 1 assigned nonrandomly:  *N* = 15, I = 8, C = 7  Community college student smokers  *Recruitment:* Announcements by college instructors, student newsletters/newspapers, school marquee announcements, and flyers. Conducted over 18 months at 15 x community colleges. | M = 22.8  SD = 4.7  F = 58.5% | I = 4 x motivational interviewing session + tailored feedback about lung function, expired carbon monoxide levels, and tailored messages about readiness to quit and smoking-related characteristics provided by computer software and delivered by counselor + summary of data in brief newsletter.  C = 4 x 5-10 minute session of brief counseling, advised to quit, copy of smoking cessation manual, at 3 x follow-up sessions, counselor enquired about quitting progress, answered questions, and provided different fact sheet for each of the 3 sessions. | Smoked at least one cigarette per day  Treatment  Stages of Change | I = Computer  C = None | I = N  C = N | I = TA  C = MC | Y = Mixed models | 6/9 | 1. Cotinine-validated 7-day abstinence from smoking  2. Self-report 7-day abstinence from smoking  Baseline  10 months  10 months – achieved 7-day abstinence, N (%):  Cotinine-validated:  I = 25 (16.6)  C = 17 (10.1)  Self-report:  I = 45 (28.5)  C = 41 (24.4) | 10 months:  1. N  2. N | 6 months:  1. 0.28  2. 0.12 |
| Spit tobacco (*n* = 1) | | | | | | | | | | | | |
| Masouredis (1997) [66] *  USA | *N* = 360, I = 171, C = 189 (spit tobacco users only analyzed);  *N* = 1208, I = 556, C = 652 (including nonusers).  16 campuses randomized  *N* = 16, I = 8, C = 8  Male baseball and football college athletes with self-reported spit tobacco use.  *Recruitment:* The first of 16 (of 35) colleges contacted and willing to participate were enrolled in the study. 1208 athletes were then screened for spit tobacco use and randomized within colleges. | M = 19.8  SD = 1.6  F = 0.0% | I = Oral examination by dentist or dental hygienist + advice to quit + self-help guide to quitting + 15-20 minutes of counseling + 2 x follow-up phone calls (at quit date and one month after intervention) by dental hygienist. Nonusers and former users also received a small group session with the dental hygienist to encourage them to support users to quit.  C = No intervention. | Self-reported spit tobacco use more than once per month or seasonal users planning to use spit tobacco more than once per month during the next season  Treatment and prevention  Health belief model & cognitive social learning theory. | I = Telephone  C = N/A | I = Combined  C = N/A | I = MC  C = N/A | Y = BOCF | 5/9 | Cotinine-validated 7-day abstinence from tobacco  Baseline  3 months  3 months – achieved 7-day abstinence, N (%):  I = 41 (24.0)  C = 30 16.0) | 3 months:  Y | 3 months:  0.28 |
| Marijuana (*n* = 2) | | | | | | | | | | | | |
| Lee (2010) [68]  USA | *N* = 341, I = 171, C = 170  Incoming students at university  *Recruitment:* Mailed letter and email containing link to screening survey to 4000 incoming students. | M = 18.03  SD = 0.31  F = 54.6% | I = Brief web-based personalized feedback about marijuana use presented in text and pictures, printable, and could view the feedback for 3 months.  C = No intervention | Any use of marijuana in 3 months prior to screening.  Treatment  Motivational Interviewing | I = internet  C = N/A | I = Y  C = N/A | I = SA  C = N/A | Y = EM | 7/9 | Marijuana use (number of days during previous 90 days)  3 months  6 months  3 months – number of days of marijuana use, M (SD):  I = 9.14 (14.07)  C = 9.06 (15.78)  6 months – number of days of marijuana use, M (SD):  I = 11.05 (18.71)  C = 11.94 (19.31) | 3 months:  N  6 months:  N  Study tested outcome over time (ANOVA) | 3 months:  -0.01  6 months:  0.05 |
| Elliott (2012)  [70]  USA | *N* = 245, I = 111, C = 134  College students  *Recruitment:* Recruited from psychology courses for class credit as part of a larger intervention study on marijuana users. | M = 20.5  SD = 2.7  F = 73.0% | I = 20-minute web-based prevention and intervention program providing assessment and feedback about perceived norms of marijuana use, other drug use, money spent on substances, etc. + suggestions for campus resources and steps for decreasing use.  C = No intervention | No marijuana use in past 30 days (abstainers)  Nonusers  None identified. | I = internet  C = N/A | I = Y  C = N/A | I = SA  C = N/A | Y = Not described | 6/9 | Any marijuana use during previous month.  1 month  1 month – any marijuana use during previous month, N (%):  I = 6 (5.5)  C = 15 (11.4) | 1 month:  N | 1 month:  0.38  (author provided) |
| Stress, marijuana, alcohol and tobacco (*n* = 1) | | | | | | | | | | | | |
| Moore et al. (2012) [69]  USA | *N* = 200, I = 102, C = 98  College students  *Recruitment:* Students approached by trained research staff in common areas on university campus. | M = 19.44  SD = 1.06  F = 51.0% | I = 1 x 20 minute session of ”Project Fitness” computer program with gain/loss-framed messages about health behaviors + screening items and tailored feedback.  C = 1 x session of public domain brochure “Fitness Fundamentals: Guidelines for Personal Exercise Programs” converted to computer program. | None  Universal  Behavior-Image model | I = Computer  C = Computer | I = N  C = N | I = SA  C = SA | Y = no dropout | 5/9 | Intentions – cigarettes  Intentions – marijuana    Baseline  post (immediate)  (no abstinence measure)  Post (immediate) –  Intentions to smoke cigarettes in next 6 months, M (SD):  I = 1.30 (0.70)  C = 1.55 (1.01)  Intentions – intentions to use marijuana in next 6 months, M (SD):  I = 1.63 (1.08)  C = 1.90 (1.17) | Post (immediate):  Y  N  Study tested outcome over time (ANOVA) | Post (immediate):  0.29  0.27 |

Notes: * = Studies included in the meta-analysis; SMS = Short Message Service (Mobile phone); SA = self-administered therapy, PSH = predominantly self-help, MC = minimal-contact therapy, TA = predominantly therapist-administered treatments, ID = Insufficient data, NC = No control group, unable to be calculated, NR = Not reported, NS = Not significant, BOCF = Baseline observation carried forward; Y = Yes, *N* = No; ELM = Elaboration Likelihood Model, EM = Expectation maximization.
